# The YAP/SERCA2a signaling pathway protects cardiomyocytes against reperfusion-induced apoptosis

**DOI:** 10.18632/aging.103481

**Published:** 2020-07-09

**Authors:** Jiankai Zhong, Haichun Ouyang, Sulin Zheng, Zhongzhou Guo, Yuying Chen, Yuanlin Zhong, Wenhao Zhong, Liuer Zuo, Jianhua Lu

**Affiliations:** 1 Department of Cardiology, Shunde Hospital, Southern Medical University (The First People’s Hospital of Shunde), Foshan 528308, Guangdong, China; 2 Department of Cardiology, Huiqiao Medical Center, Nanfang Hospital, Southern Medical University, Guangzhou 510515, Guangdong, China; 3 Department of Intensive Care Unit, Shunde Hospital, Southern Medical University (The First People’s Hospital of Shunde), Foshan 528308, Guangdong, China

**Keywords:** SERCA2a, YAP, cardiomyocytes, I/R injury, mitochondrial, ER

## Abstract

Mitochondria and the endoplasmic reticulum (ER) are known to promote cardiac ischemia/reperfusion (I/R) injury. Overexpression of yes-associated protein (YAP) and/or sarcoplasmic reticulum calcium ATPase 2a (SERCA2a) has been shown to protect cardiomyocytes against I/R-induced injury. Here, we show that activation of the YAP/SERCA2a pathway attenuated mitochondrial damage and ER stress (ERS) to maintain cardiomyocyte viability in the setting of I/R injury. Our results demonstrate that I/R treatment reduced the transcription and expression of *YAP* and *SERCA2a*, along with a decline in cardiomyocyte viability. The overexpression of *YAP* promoted *SERCA2a* transcription, whereas *SERCA2a* upregulation did not affect the *YAP* transcription, suggesting that YAP functions upstream of SERCA2a. Activation of the YAP/SERCA2a pathway suppressed mitochondrial damage by sustaining the mitochondrial redox balance and restoring mitochondrial bioenergetics. Additionally, its activation repressed ERS, reduced calcium overload, and eventually blocked caspase activation. The knockdown of *SERCA2a* suppressed the protective effects of *YAP* overexpression on mitochondrial damage and ERS. Overall, our findings reveal that the YAP/SERCA2a pathway attenuates the mitochondrial damage and ERS in response to cardiac I/R injury by regulating the mitochondria–ER communication.

## INTRODUCTION

Mitochondrial dysfunction and endoplasmic reticulum (ER) stress following ischemia/reperfusion (I/R) injury contribute to the pathogenic processes underlying cardiac damage [1, 2]. The I/R injury-induced excessive production of reactive oxygen species (ROS) within mitochondria activates the apoptotic pathway, leading to cardiomyocyte death and tissue injury [3, 4]. In addition, oxidative stress, produced by mitochondrial ROS accumulation, causes unfolding of proteins within the ER, resulting in ER stress (ERS) [5, 6]. As the heart is highly susceptible to I/R injury, the prevention of ischemic or reperfusion-induced events has become an important aspect of the modern cardiac health care system.

The association between ER and mitochondria—the ER–mitochondrial interface—is a highly dynamic structure that transports calcium, lipids, ROS, and small proteins between mitochondria and ER, as well as coordinates several intracellular processes and signaling pathways [7–9]. Studies by our group [10, 11] and others [12, 13] report that the ER–mitochondrial interface functions in several post-I/R events, including cardiomyocyte death, inflammation response, fibrosis, and angiogenesis [12, 13]. For example, the prolonged influx of misfolded or unfolded proteins into the ER activates ERS-related signaling cascades to clear these abnormal proteins [14, 15]. Moreover, ERS induces apoptosis via the intrinsic pathway involving mitochondria, further contributing to the I/R damage in myocardial tissues [18–21]. Bax-induced hyper-permeabilization of the mitochondrial outer membrane releases pro-apoptotic factors, such as cytochrome c, from the mitochondria into the cytosol to trigger apoptosis [16]. In addition, mitochondrial fission, triggered by Drp1 recruitment from the cytoplasm to the surface of mitochondria, is involved in mitochondrial outer membrane remodeling and mitochondrial apoptotic pathway activation [17–19]. Interestingly, Drp1-induced mitochondrial fission is controlled by ER, which recruits Drp1 to potential division sites [20].

Sarcoplasmic reticulum calcium ATPase 2° (SERCA2a) regulates the functions of ER and mitochondria [21]. Increased SERCA2a expression reduces the calcium levels in mitochondria via calcium reflux into the ER [21]. Decreased intracellular calcium effectively restores the expression and activity of anti-oxidative factors, thus suppressing oxidative stress [21]. Restoration of normal redox biology reduces the levels of oxidized proteins or unfolded peptides, ameliorating the ERS in cardiomyocytes [22, 23]. These findings were first reported in hyperglycemia-treated cardiomyocytes but not in the context of cardiac I/R injury.

The Hippo pathway, found both in *Drosophila* and mammals, contains a core kinase cassette comprising mammalian STE20-like protein kinases 1 and 2 (MST1/2; homologs of Hpo in *D. melanogaster*); large tumor suppressor kinases 1 and 2 (LATS1/2; homologs of Wts); and their respective adaptor proteins, Salvador 1 (SAV1; a homolog of Sav), MOB kinase activators 1A and 1B (MOBKL1A/1B; homologs of Mats), and the downstream effectors of the Hippo signaling pathway-transcriptional coactivators YAP and TAZ (the two homologs of Yorkie; YAP, encoded by *YAP1*; TAZ, also known as WWTR1) [24, 25]. YAP directly targets several mitochondria-associated genes including *Drp1*, *JNK*, *ERK*, and *AMPK* [25]. Yu et al. [26] demonstrated that overexpressed YAP in transgenic mice with septic cardiomyopathy attenuated lipopolysaccharide (LPS)-induced myocardial injury and cardiac dysfunction by inhibiting mitochondrial fission in a MAPK–ERK pathway-dependent manner. Ma et al. [27] reported that the YAP–Hippo pathway attenuated the hypoxia-induced suppression of OPA1-related mitochondrial fusion both *in vivo* and *in vitro*. In the present study, we investigated whether YAP functions as the upstream transcriptional modulator of SERCA2a and affects the mitochondrial performance and ERS.

## RESULTS

### Overexpression of *YAP* or *SERCA2a* attenuates I/R-induced cardiomyocyte apoptosis

**Figure 1:**
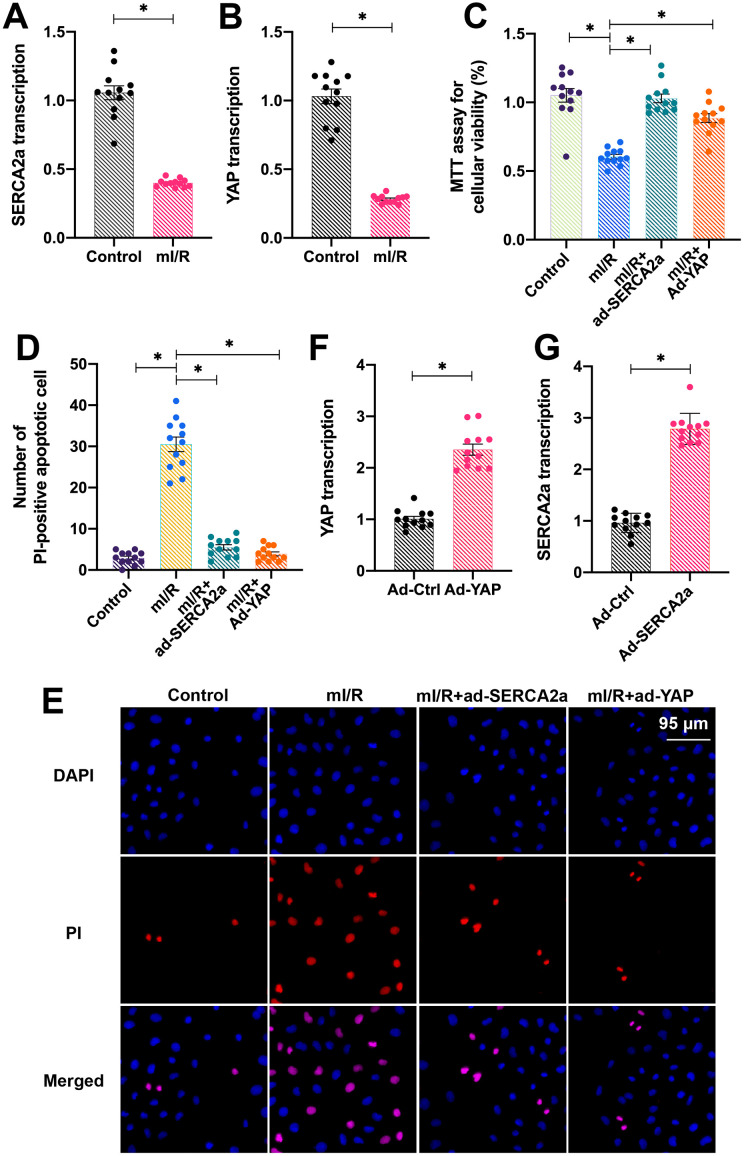
**Overexpression of *YAP* or *SERCA2a* attenuates I/R-induced cardiomyocyte apoptosis.** (**A**, **B**) Quantitative polymerase chain reaction (qPCR) assay was used to analyze the mRNA levels of YAP and SERCA2a in cardiomyocytes subjected to mI/R injury. SERCA2a adenovirus (ad-SERCA2a) and YAP adenovirus (ad-YAP) were transfected into cardiomyocytes to overexpress *SERCA2a* and *YAP*, respectively. (**C**) The MTT assay was used to detect the cardiomyocyte viability and (**D**, **E**) PI staining was used to assess the number (percentage) of apoptotic cells. (**F**, **G**) The qPCR assay was used to analyze the mRNA levels of YAP and SERCA2a in cardiomyocytes transfected with ad-SERCA2a or ad-YAP under physiological conditions. *P<0.05.

To study the role of overexpressed *YAP* and *SERCA2a* in protecting cardiomyocytes against I/R injury, isolated cardiomyocytes were cultured under hypoxic conditions for 2 h and subsequently reoxygenated for 2 h to establish an *in vitro* mimicked I/R injury (mI/R) model. Next, the total RNA was isolated and the endogenous mRNA levels of YAP and SERCA2a were determined. As shown in Figure 1A, 1B, compared with the control group, the mRNA levels of YAP and SERCA2a were downregulated in response to mI/R injury. To understand the role of SERCA2a and YAP in the setting of cardiac I/R injury, recombinant adenoviruses overexpressing *SERCA2a* (ad-SERCA2a) and *YAP* (ad-YAP) were transfected into cardiomyocytes before mI/R injury. Next, the cardiomyocyte viability and apoptotic rate were measured. As shown in Figure 1C, compared with the control group, the overexpression of *SERCA2a* or *YAP* decreased the mI/R injury-induced apoptosis. In addition, propidium iodide (PI) staining demonstrated a reduced number of apoptotic cardiomyocytes after transfection with either ad-SERCA2a or ad-YAP (Figure 1D, 1E). The overexpression efficiency was confirmed by quantitative polymerase chain reaction (qPCR) (Figure 1F, 1G). Altogether, our results indicated that overexpression of *YAP* or *SERCA2a* attenuated the mI/R injury-induced cardiomyocyte apoptosis.

### SERCA2a is transcriptionally regulated by YAP in cardiomyocytes

**Figure 2:**
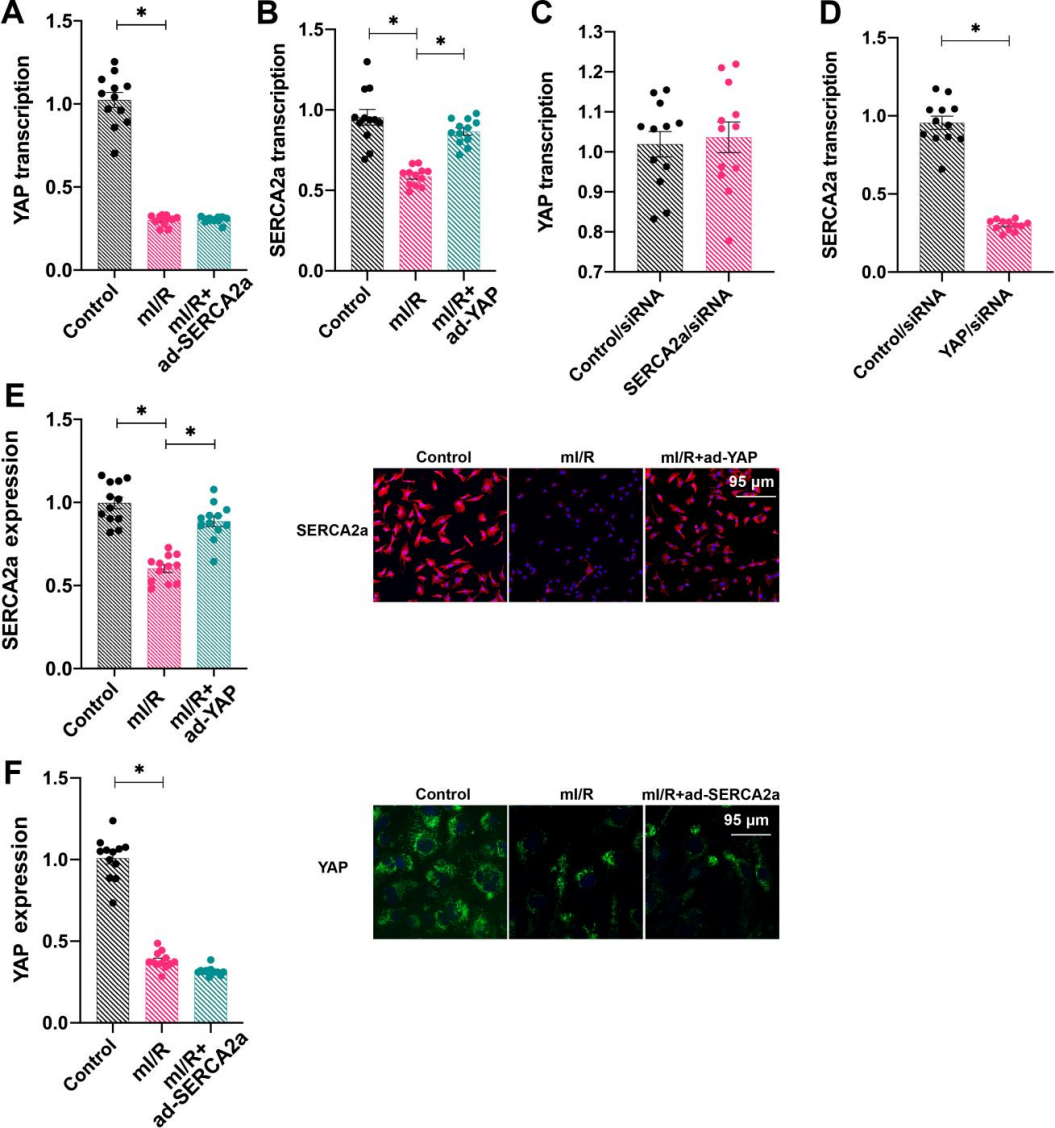
***SERCA2a* is transcriptionally regulated by YAP in cardiomyocytes.** (**A**, **B**) Cardiomyocytes were transfected with ad-SERCA2a and ad-YAP to overexpress *SERCA2a* and *YAP*, respectively. Quantitative polymerase chain reaction (qPCR) assay was used to analyze the mRNA levels of YAP and SERCA2a in cardiomyocytes subjected to mI/R injury. (**C**, **D**) The qPCR assay was used to detect the mRNA levels of YAP and SERCA2a in cardiomyocytes transfected with *SERCA2a* siRNA or *YAP* siRNA. (**E**, **F**) Immunofluorescence assay was used to detect the expression of SERCA2a and YAP in cardiomyocytes subjected to mI/R using anti-SERCA2a (pink) and anti-YAP (green) antibodies, respectively. Scale bars, 95 μm. Left panels show quantification of the expression of SERCA2a and YAP.*P<0.05.

To understand the relationship between SERCA2a and YAP, ad-SERCA2a and ad-YAP were transfected into cardiomyocytes under mI/R setting and their mRNA levels were measured. As shown in Figure 2A, 2B, ad-YAP transfection increased the transcription of *SERCA2a*, whereas ad-SERCA2a had no effect on *YAP* transcription, suggesting that *YAP* overexpression promoted the SERCA2a translation. To confirm this finding, *SERCA2a* siRNA and *YAP* siRNA were transfected into cardiomyocytes under normal conditions. The silencing of *SERCA2a* had no effect on *YAP* transcription; however, *YAP* knockdown reduced the transcription of *SERCA2a* (Figure 2C, 2D). In addition, immunofluorescence assays demonstrated that the protein expression of SERCA2a was downregulated in response to mI/R injury, whereas ad-YAP transfection increased its expression (Figure 2E, 2F). In contrast, ad-SERCA2a overexpression had no marked effect on the protein expression of YAP in mI/R-treated cardiomyocytes (Figure 2E, 2F). Altogether, our results indicated that SERCA2a was transcriptionally regulated by YAP.

### Activation of the YAP/SERCA2a pathway reduces mitochondrial damage in I/R-treated cardiomyocytes

**Figure 3:**
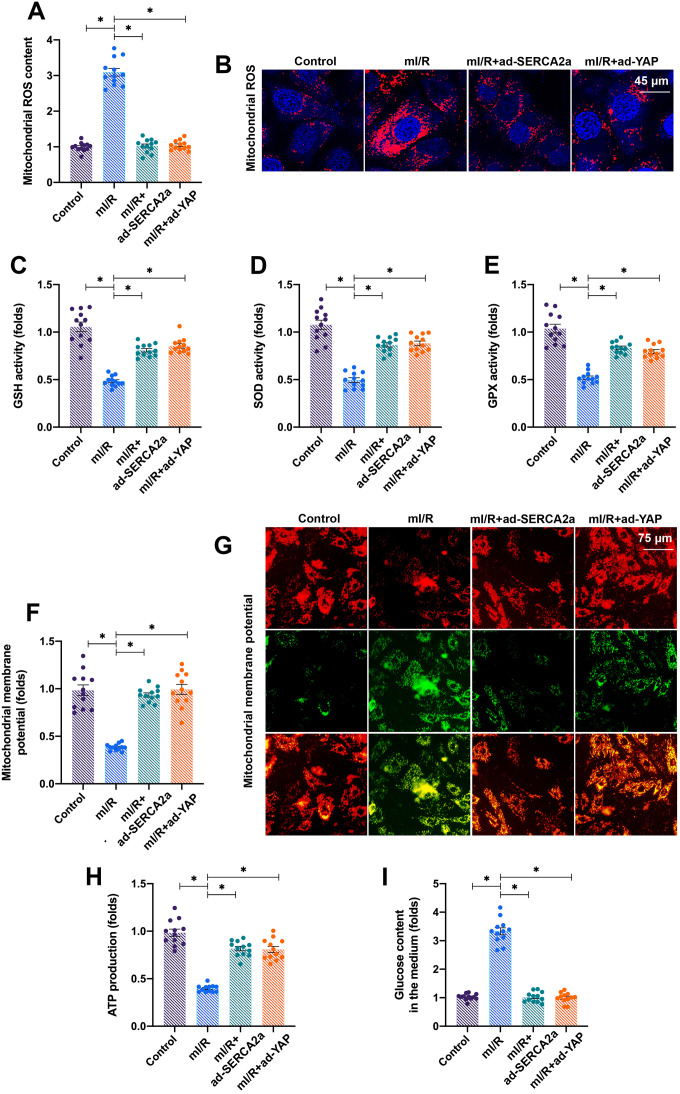
**Activation of the YAP/SERCA2a pathway reduces mitochondrial damage in I/R-treated cardiomyocytes.** (**A**, **B**) Immunofluorescence assay was used to detect the levels of mitochondrial ROS in cardiomyocytes transfected with ad-SERCA2a or ad-YAP in the presence of mI/R injury. (**C**–**E**) The activities of glutathione (GSH), superoxide dismutase (SOD), and glutathione peroxidase (GPx) were measured via enzyme-linked immunosorbent assay (ELISA) in cardiomyocytes transfected with ad-SERCA2a or ad-YAP in the presence of mI/R injury. (**F**, **G**) Mitochondrial membrane potential was measured using JC-1 staining. (**H**) ATP production was analyzed through ELISA. (**I**) The levels of glucose in the medium were determined by ELISA. *P<0.05.

We next investigated how the YAP/SERCA2a pathway protected cardiomyocytes against mI/R injury. Several previous studies have suggested that mitochondria and ER are the two primary targets for reperfusion-induced myocardial injury [16, 21]. Thus, we focused on the alterations in mitochondrial function in response to *YAP* and/or *SERCA2a* overexpression. The levels of mitochondrial ROS rapidly increased after mI/R injury (Figure 3A, 3B), indicating oxidative stress. Interestingly, the overexpression of *YAP* or *SERCA2a* reduced the levels of mitochondrial ROS (Figure 3A, 3B). In addition, the levels of anti-oxidative factors, such as glutathione (GSH), superoxide dismutase (SOD), and glutathione peroxidase (GPx), which rapidly decreased following mI/R injury, were elevated after overexpression of *YAP* or *SERCA2a* (Figure 3C–3E). These results indicated that the mitochondrial redox balance was sustained by SERCA2a and YAP in cardiomyocytes.

In addition to oxidative stress, we observed that the mI/R injury reduced the mitochondrial membrane potential, which was reversed by *YAP* or *SERCA2a* overexpression (Figure 3F, 3G). The mitochondrial membrane potential acts as the driving force for cellular ATP production by controlling glucose consumption. The mI/R injury inhibited ATP production in cardiomyocytes (Figure 3H), followed by an accumulation of glucose in the culture medium (Figure 3I). The overexpression of *YAP* or *SERCA2a* enhanced the consumption of glucose, thus sustaining ATP production (Figure 3H, 3I). Altogether, our results showed that YAP and SERCA2a controlled the mitochondrial redox balance and bioenergetics in mI/R-treated cardiomyocytes.

### Activation of the YAP/SERCA2a pathway attenuates ERS in I/R-treated cardiomyocytes

**Figure 4:**
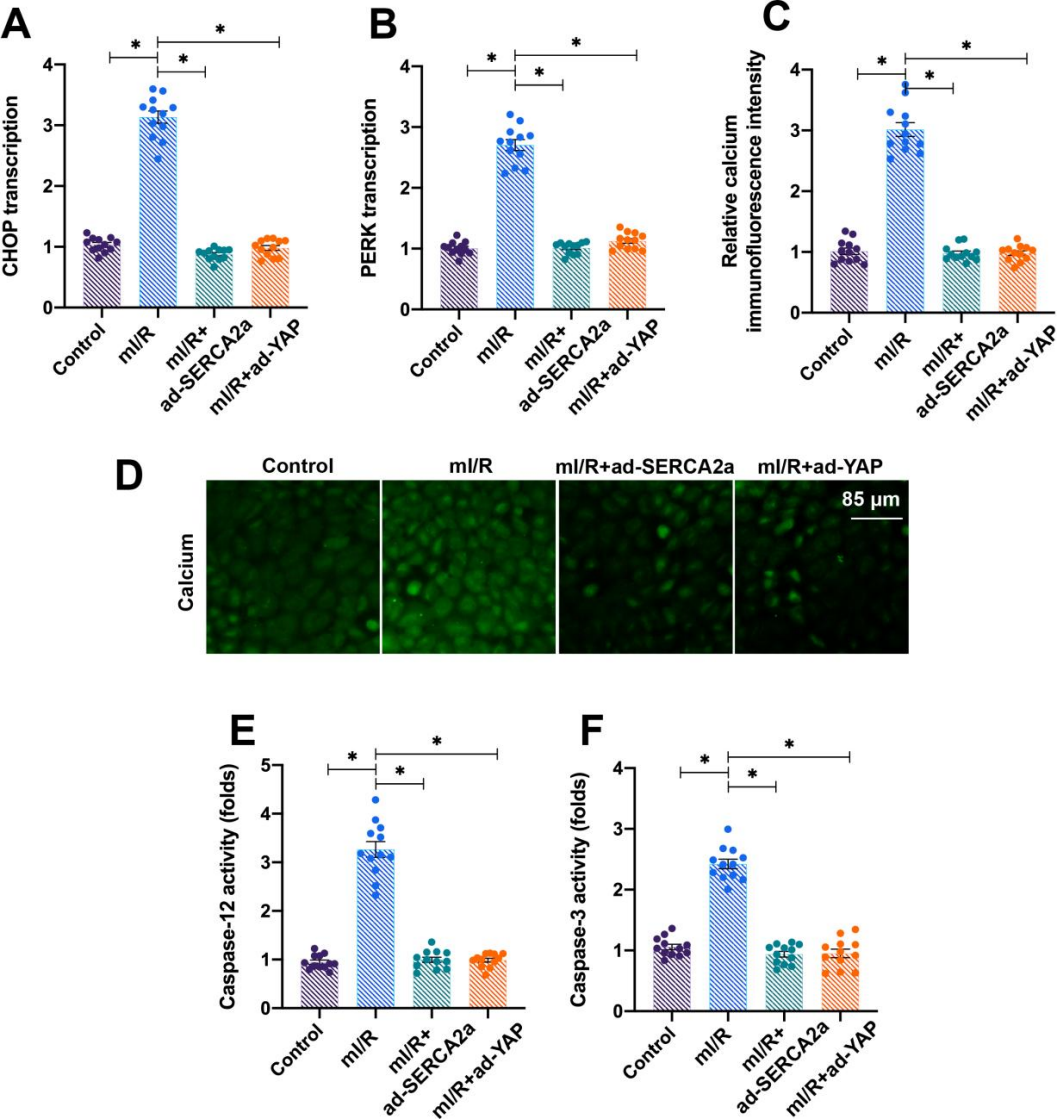
**Activation of the YAP/SERCA2a pathway attenuates ERS in I/R-treated cardiomyocytes.** (**A**, **B**) Cardiomyocytes were transfected with ad-SERCA2a and ad-YAP to overexpress *SERCA2a* and *YAP*, respectively. Quantitative polymerase chain reaction (qPCR) assay was used to analyze the mRNA levels of PERK and CHOP in cardiomyocytes transfected with ad-SERCA2a or ad-YAP in the presence of mI/R injury. (**C**) Quantification of relative calcium immunofluorescence intensity in cardiomyocytes transfected with ad-SERCA2a or ad-YAP in the presence of mI/R injury (**D**) Immunofluorescence assay was used to assess the baseline calcium overload in cardiomyocytes. (**E**, **F**) Enzyme-linked immunosorbent assay (ELISA) was used to detect the activation of caspase-12 and caspase-3. Scale bar, 85 μm.*P<0.05.

Mitochondria are the centers of cellular energy metabolism, and ER acts as the manufactory for protein synthesis [28]. Under physiological conditions, cells employ ERS, a process activated by the accumulation of unfolded proteins, for rapid protein turnover [29]. In the present study, we found that the mI/R injury activated ERS in cardiomyocytes, as evidenced by increased mRNA levels of PREK and CHOP (Figure 4A, 4B). Interestingly, the overexpression of *YAP* or *SERCA2a* repressed the ERS in mI/R-treated cardiomyocytes.

Furthermore, ER maintains the intracellular calcium balance by regulating the activity of calcium channels, such as IP3R and RyR, localized on its surface [22, 30]. In the present study, we found that the levels of intracellular calcium increased after exposure of cardiomyocytes to mI/R injury (Figure 4C, 4D), indicating baseline calcium overload. The activities of apoptotic proteins caspase-12 and caspase-3 increased following mI/R injury, indicating that apoptosis was triggered by ERS (Figure 4E, 4F). Interestingly, the overexpression of *YAP* or *SERCA2a* attenuated the calcium overload (Figure 4C, 4D) and reduced the activity of caspase-12 and caspase-3 (Figure 4E, 4F). Altogether, our results demonstrated that the activation of YAP/SERCA2a attenuated ERS in cardiomyocytes.

### Knockdown of *SERCA2a* attenuates YAP-induced protection on I/R-treated cardiomyocytes

**Figure 5:**
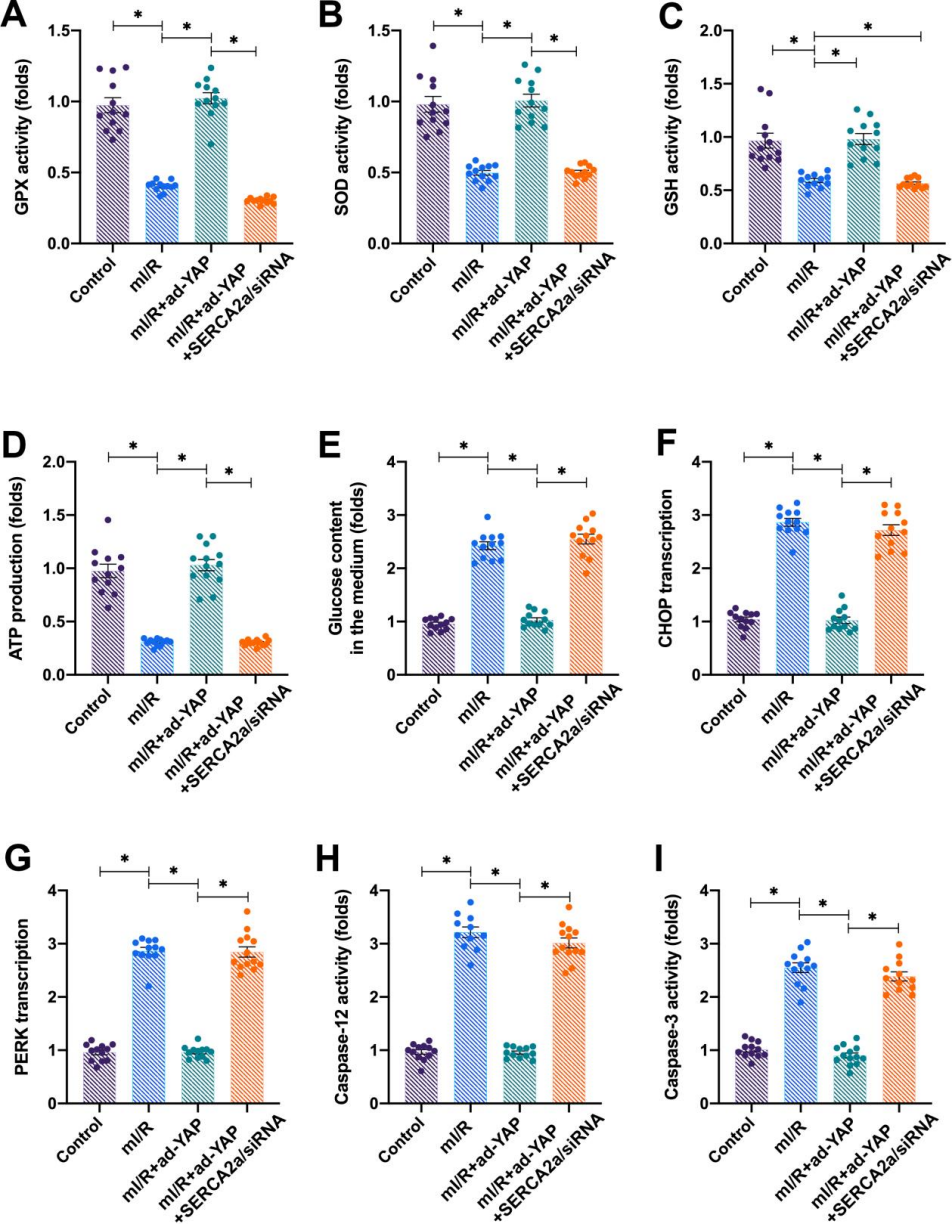
**Knockdown of *SERCA2a* attenuates YAP-induced protective effects on mitochondria and ERS in I/R-treated cardiomyocytes.** *SERCA2a* siRNA was transfected into cardiomyocytes infected with ad-YAP in the presence of mI/R injury. (**A**–**C**) The activity of glutathione (GSH), superoxide dismutase (SOD), and glutathione peroxidase (GPx) were measured via enzyme-linked immunosorbent assay (ELISA). (**D**) ATP production was determined through ELISA. (**E**) The levels of glucose in the medium were determined through ELISA. (**F**, **G**) Quantitative polymerase chain reaction (qPCR) assay was used to analyze the mRNA levels of PERK and CHOP. (**H**, **I**) ELISA was used to detect the activation of caspase-12 and caspase-3. *P<0.05.

To verify whether YAP-induced cardiac protection was dependent on SERCA2a, *SERCA2a* siRNA and ad-YA were co-transfected into cardiomyocytes. Next, mitochondrial function and ERS were measured. Although the overexpression of *YAP* attenuated the mI/R-induced mitochondrial oxidative stress (Figure 5A–5C), this effect was suppressed by co-transfection with *SERCA2a* siRNA. In addition, the overexpression of *YAP* sustained the mitochondrial ATP production and glucose consumption (Figure 5D, 5E); these effects were negated by *SERCA2a* siRNA co-transfection. In mI/R-treated cardiomyocytes, *YAP* overexpression suppressed the mRNA levels of CHOP and PERK, whereas this effect was negated in cardiomyocytes co-transfected with *SERCA2a* siRNA (Figure 5F, 5G). Similarly, mI/R-induced caspase-12 and caspase-3 activation was suppressed by *YAP* overexpression, whereas *SERCA2a* siRNA suppressed the inhibitory action of YAP on caspase-3 and caspase-12 (Figure 5H, 5I). Altogether, our results indicated that YAP protected cardiomyocytes from mitochondrial damage and ER stress following I/R injury in a SERCA2a-dependent manner.

## DISCUSSION

According to the ACCF/AHA/SCAI guidelines, early revascularization is required to control myocardial injury, limb injury, and complications during organ transplantation. However, optimal clinical outcomes have not been reported after the restoration of blood flow to the ischemic tissue [31]. Post-ischemic damage or I/R injury significantly compromises the clinical benefits of revascularization strategies [32]. Previous studies have reported the involvement of mitochondria and ER in the progression of cardiac I/R injury [33, 34]. Damaged mitochondria fail to supply sufficient ATP required for cardiomyocyte contraction, leading to myocardial depression [35, 36]. Impaired ER function or ERS activates the members of the caspase family, triggering apoptosis of cardiomyocytes [37, 38]. Therefore, understanding the link between mitochondrial damage and ERS during cardiac I/R injury is vital for the design and development of novel drugs or therapeutic approaches for patients undergoing revascularization. In this study, we suggest that the YAP/SERCA2a signaling pathway functions upstream of mitochondrial damage and ERS in I/R-challenged cardiomyocytes. Exposure to I/R injury downregulated the expression of *YAP* and *SERCA2a*, and reduced cardiomyocyte viability. Overexpression of *YAP* transcriptionally upregulated SERCA2a expression and sustained mitochondrial function and ER homoeostasis. These findings identified the YAP/SERCA2a axis to simultaneously protect mitochondria and ER in I/R-treated cardiomyocytes. Therefore, developing therapeutic approaches that target the YAP/SERCA2a pathway would clinically benefit patients with cardiac I/R injury.

The hearts of mammals, including humans, have a poor regenerative capacity. Cardiovascular disorders in most patients advance to heart failure [39], ultimately leading to death. Inactivation of YAP in the myocardium of adult mice improved cell survival and cardiac function after myocardial infarction [40]. Moreover, a study reported that YAP activation reversed mitophagy and promoted mitochondrial fission, both induced by oxidative stress, to attenuate cardiac dysfunction after I/R injury [26]. Similarly, phosphorylation of YAP activated the Hippo pathway in mice with diabetic cardiomyopathy, increasing cardiomyocyte contractility and reducing cardiac injury by coordinating mitophagy and apoptosis [41]. In mice with septic cardiomyopathy, inhibition of YAP augmented cardiomyocyte damage by altering the mitochondrial quality control system under inflammatory stress [26]. Several drugs are used to target the YAP–Hippo signaling pathway in different kinds of cancers [42, 43]; however, few medicines are available for cardiovascular disorders. We report YAP as a transcriptional factor that promotes the expression of *SERCA2a* in I/R-treated cardiomyocytes [44]. Previous studies have described a direct role of YAP in regulating cardiomyocyte viability. Our study identifies the downstream effectors of YAP and reveals how YAP indirectly acts on them. More studies are required to explore how YAP binds to the promoter of *SERCA2a* and control its mRNA levels.

SERCA2a is an ATP-dependent protein kinase that utilizes mitochondrial ATP to maintain calcium homeostasis during the cardiac cycle. It controls the transport of cytoplasmic calcium to the ER and thus prevents ERS. We have previously reported that both the expression and activity of *SERCA2a* were downregulated in cardiomyocytes following I/R injury [45]. In the present study, we report that SERCA2a is transcriptionally regulated by YAP, such that reduced levels of YAP cause SERCA2a downregulation in I/R-treated cardiomyocytes. In addition, a recent study has reported that HAX-1 regulates post-transcriptional modifications, including oxidation and degradation, of SERCA2a in cardiomyocytes [46]. SERCA2a downregulation or inactivation is closely associated with myocardial infarction [47], atrial arrhythmias [48], hypertension [49], cardiac aging [50], heart failure [51], and myocardial hypertrophy [52]. We show that SERCA2a sustains mitochondrial function and ER homeostasis by promoting mitochondrial metabolism, maintaining intracellular calcium homeostasis and preventing ERS.

In conclusion, cardiac I/R injury promotes *YAP* downregulation and SERCA2a inactivation. Activation of the YAP/SERCA2a pathway sustains cardiomyocyte viability and function by attenuating I/R injury-induced mitochondrial damage and ERS. These findings highlight that the dysregulated YAP/SERCA2a pathway could be involved in the pathology underlying cardiac I/R injury in a mitochondria and ER-dependent manner.

## MATERIALS AND METHODS

### Isolation and primary culture of mouse cardiomyocytes

Cardiomyocytes were isolated from 1- to 3-day old C57BL/6 mice according to the procedures described in our previous studies [23, 53]. In brief, after the heart tissue was dissected and washed, it was finely minced and the chunks were placed in 0.25% trypsin. The pooled cell suspension was centrifuged and resuspended in Dulbecco’s modified Eagle’s medium (DMEM) supplemented with 10% fetal bovine serum, 100 U/mL penicillin, and 100 μg/mL streptomycin. The resuspension was cultured in a tissue culture flask for 90 min at 37°C to allow the fibroblasts to attach to the flask bottom [54]. The non-adherent and weakly attached cells, mainly cardiomyocytes, were removed and seeded into culture plates. Next, 5-bromo-2’-deoxyuridine (5-BrdU, 10 nM; #B5002; Sigma, Saint Louis, USA) was added to the culture medium to remove fibroblasts. The cells were incubated at 37°C in a humidified atmosphere containing 5% CO_2_ and 95% air. After 48 h, cardiomyocytes that adhered to the culture dish were used for subsequent experiments [55]. Cells were deprived of serum and placed in an anoxic chamber for 12 h in a humidified atmosphere comprising 5% CO_2_ and 95% N_2_.

### Protein extraction and western blotting

Total proteins were extracted from cardiac tissue using the T-PER extraction reagent (Thermo Scientific) [56]. All cell extracts were prepared using lysis buffer that included the following: 50 mM Tris (pH 7.5), 120 mmol/L NaCl, 6 mmol/L EGTA, 1 mmol/L EDTA, 20 mmol/L NaF, 1 mmol/L sodium pyrophosphate, 30 mmol/L 4-nitrophenyl phosphate, 0.1% Nonidet P-40, 1 mmol/L benzamidine, and a protease inhibitor mixture (Roche). The extracted proteins were resolved on 10% SDS PAGE and electroblotted onto PVDF membranes (Bio-Rad). After blocking the membranes in TBST (Tris-buffered saline with Tween 20) containing 5% [w/v] non-fat dry milk for 1 h at room temperature, these were washed thrice [57], and incubated overnight with primary antibodies diluted at optimal concentrations in 5% non-fat milk solution. The membranes were washed and incubated with a donkey anti-rabbit or anti-mouse IRDye-conjugated IgG (Li-Cor Odyssey) secondary antibody (dilution 1:3000) for 1 h [58]. The blots were scanned, and the images were displayed in grayscale. The intensity of the protein bands was quantified using an image processing program (Li-Cor Odyssey).

### Construction of adenoviruses

To construct adenoviral vectors overexpressing *SERCA2a* and *YAP* genes, the DNA sequences encoding for these genes were PCR amplified using high-fidelity Pfu polymerase (Agilent Technologies). PCR products were sequenced and cloned into the vector AdTrack-CMV (Agilent Technologies) [59]. Next, complete genomes of recombinant adenoviruses were constructed by homologous recombination between the recombinant AdTrack-CMV and the Ad-Easy vectors in *Escherichia coli* [60]. The adenoviruses were packaged in HEK293 cells and purified by CsCl_2_ density gradient ultracentrifugation. Adenovirus genomic DNA was purified using the NucleoSpin virus kit (Macherey-Nagel), and adenoviral titers were determined using the Adeno-X qPCR titration kit (632252, Clontech).

### siRNA-induced gene knockdown

Cardiomyocytes were transfected with *SERCA2a* siRNA or non-targeting siRNA (negative control #1 siRNA, Thermo Fisher Scientific) using Lipofectamine RNAiMAX reagent (13778150, Thermo Fisher Scientific) according to the manufacturer’s instructions [61]. The silencing of SERCA2a miRNA was confirmed through qPCR [62].

### Immunostaining

Cells were seeded onto collagen type I-coated cell culture chamber slides, fixed with 4% paraformaldehyde, and permeabilized using 0.3% Triton-X 100 [63]. JC-1 probe (Beyotime, China, Cat. No:C2006) was used to detect mitochondrial membrane potential. The cells were incubated with primary antibodies followed by incubation with Alexa Fluor secondary antibodies (1:1000). Coverslips were mounted onto slides using the ProLong Gold Antifade Mountant containing DAPI (Life Technologies) [64], and images were acquired with an Olympus IX73 microscope. Appropriate negative controls were used to perform background correction. Five fields/muscle section or culture slide were randomly counted, and all quantifications were performed by investigators blinded to the treatment [65].

### TUNEL assay

The samples were fixed with 4% paraformaldehyde and analyzed for apoptosis by terminal deoxynucleotidyl transferase dUTP nick-end labeling (TUNEL) assay [66]. The assay was performed using the ApopTag peroxidase in situ apoptosis detection kit (S7100; EMD Millipore, CA, USA) according to the manufacturer’s instructions [67].

### Measurement of intracellular calcium

Cardiomyocytes were transfected with siRNA against *SERCA2a* and after 48 h, approximately 2 × 10^6^ cells were infected with adenovirus. After incubation with serum-free M199 for 4 h, intracellular calcium was detected using the Fluo-8 calcium flux assay kit (Abcam, ab112129) according to the manufacturer’s instructions [68]. In brief, the cells were loaded with calcium-sensitive probe Fluo-8 in Hanks’ buffer with 20 mM Hepes (HHBS) for 30 min at 37°C [69]. The cells were washed with HHBS twice. Afterward, the cells were excited at 490 nm and Ca^2+^-bound Fluo-8 emission was recorded at 525 nm.

### Statistical analysis

Data were statistically analyzed using Student’s *t* test or one-way analysis of variance (ANOVA), followed by Newman–Keuls test. Quantitative data are expressed as mean ± standard error of mean (SEM). Non-quantitative data are representative of at least three independent experiments. A P-value less than 0.05 was considered significant.

### Availability of data and materials

All data generated or analyzed during this study are included in this article.
